# Retrieval and Ranking of Combining Ontology and Content Attributes for Scientific Document

**DOI:** 10.3390/e24060810

**Published:** 2022-06-10

**Authors:** Xinyu Jiang, Bingjie Tian, Xuedong Tian

**Affiliations:** 1 School of Cyber Security and Computer, Hebei University, Baoding 071002, China; jiangxinyu919@gmail.com; 2 Hebei Machine Vision Engineering Research Center, Hebei University, Baoding 071002, China; 3 Institute of Intelligent Image and Document Information Processing, Hebei University, Baoding 071002, China; 4 International Education College, Hebei Finance University, Baoding 071051, China; tianbingjie@hbfu.edu.cn

**Keywords:** scientific document retrieval and ranking, mathematical expressions, ontology attributes, HFS, BiLSTM

## Abstract

Traditional mathematical search models retrieve scientific documents only by mathematical expressions and their contexts and do not consider the ontological attributes of scientific documents, which result in gaps between the queries and the retrieval results. To solve this problem, a retrieval and ranking model is constructed that synthesizes the information of mathematical expressions with related texts, and the ontology attributes of scientific documents are extracted to further sort the retrieval results. First, the hesitant fuzzy set of mathematical expressions is constructed by using the characteristics of the hesitant fuzzy set to address the multi-attribute problem of mathematical expression matching; then, the similarity of the mathematical expression context sentence is calculated by using the BiLSTM two-way coding feature, and the retrieval result is obtained by synthesizing the similarity between the mathematical expression and the sentence; finally, considering the ontological attributes of scientific documents, the retrieval results are ranked to obtain the final search results. The MAP_10 value of the mathematical expression retrieval results on the Ntcir-Mathir-Wikipedia-Corpus dataset is 0.815, and the average value of the NDCG@10 of the scientific document ranking results is 0.9; these results prove the effectiveness of the scientific document retrieval and ranking method.

## 1. Introduction

With the development of the internet, information has exploded rapidly, and more and more scientific documents containing many mathematical expressions have rapidly been provided. The influx of various scientific documents has made it increasingly difficult to find useful information from them. Mathematical expressions are an important component of scientific documents for describing scientific content. Therefore, retrieving scientific documents by employing mathematical expressions such as query expression have become a necessary way for researchers to find the scientific information they need [1]. However, the traditional search engines designed for full text retrieval cannot work well on the math queries, because of the special characteristics of mathematical expressions. Therefore, it is necessary to use mathematical expressions as the main subject for scientific document retrieval.

### 1.1. Related Work

At present, text-based scientific document retrieval technology is largely mature [2,3,4]. However, mathematical expression-based retrieval is still under development. In recent years, many researchers have made progress in retrieving mathematical expressions. Three main approaches have been applied for mathematical expressions retrieval in previous models: (a) Operator trees (OPTs): which captures mathematical expression appearance [5]. For instance, Zhong et al. [6] proposed a dynamic pruning algorithm to solve the substructure retrieval of mathematical expressions. This retrieval algorithm expresses the mathematical expression as OPTs, which improves the efficiency of the mathematical expression retrieval. (b) Symbol layout trees (SLTs): which captures mathematical expression syntax [7,8]. (c) Embedding models: which converts two-dimensional mathematical expressions into one-dimensional vectors by using word embedding models [9,10,11,12].

Mathematical expressions have a complex two-dimensional structure, so it is very reasonable to introduce multi-criteria decision-making (MCDM) theory in the retrieval of mathematical expressions. MCDM theory has made many advances in recent years [13,14,15], and has been applied and has achieved good results in location of a fleet [16], the selection of warships [17], and information security risk assessment in critical infrastructure [18]. Hesitant fuzzy sets (HFSs) are one of the MCDM theories, and as an extension of fuzzy sets, they have made achievements in theory as well as in numerous other fields [19,20]. HFSs have been proven as a potential structure to express the uncertainty and vagueness [21,22], which can measure the impact of each attribute on decision making in an integrated way and are more flexible in expressing hesitant information in terms of processing. Driven by their unique advantages and the richness of their applications, we find them applicable to the retrieval of mathematical expressions with multiple attributes.

In terms of text similarity, Bromley et al. [23] first proposed the Siamese network in 1993, and its model has the parameter-sharing property, which is very suitable for calculating sentences’ similarity. Therefore, Wang et al. [24] proposed a bilateral multi-perspective matching model. They used the bidirectional LSTM combined with the Siamese network. The sentences are bilaterally encoded and matched in multiple ways to obtain the final sentence similarity, and the introduction of bilateral and multi-perspective matching makes the model more able to capture the semantic information of sentences. Liu et al. [25] proposed a sentence similarity model with multi-feature fusion, introduced syntactic structure and word order features, and improved the accuracy of sentence similarity.

In the comprehensive retrieval of mathematical expressions and text, Zhong et al. [26] used an improved OPT algorithm for retrieval of mathematical expressions and mined contextual potential keywords as query extensions, which explored the semantics of mathematical expressions and enabled a more accurate retrieval of relevant mathematical content. Kristianto et al. [27] proposed a dependency graph method to enrich the semantic information of mathematical expressions because of the difficulty of capturing the semantics of mathematical expression context, and the experimental results showed that the accuracy of the mathematical search system can be improved by 13%. Tian et al. [28] proposed a scientific document retrieval method based on the hesitant fuzzy set and BERT. They first used the hesitant fuzzy set to retrieve the mathematical expression, then used BERT to encode the keywords into word vectors, and finally used the cosine similarity to calculate the similarity between two keywords. On this basis, Tian et al. [29] extracted full-text keywords, and then the GBDT model was used to discrete and reorganize mathematical expressions and text attributes; finally, the LR model was used to train the attributes to obtain the final retrieval results. The results showed that the comprehensive mathematical expression and the context of the scientific document retrieval were more reasonable. Pathak et al. [30,31] designed a knowledge base (KB) containing contextual formula pairs, and a total of 12,573 pairs of formulas and their contexts were extracted, considering the similarity between mathematical expressions, contexts, and documents. This method considered the relationship between the mathematical expression itself and its context, and then made the retrieval more credible. In 2019, Yuan et al. [32] proposed a new abstract model based on the mathematical content “MathSum”, which uses the pointer mechanism and the multi-head attention mechanism to extract the mathematical content of the text and enrich the semantics of mathematical expressions, respectively, which provide new ideas for retrieving scientific documents. In 2019, Dhar et al. [33] proposed a signature-based hashing scheme, which constructed the search engine “SigMa”, based on mathematical expressions, to retrieve documents by perceiving the high structure in mathematical expressions, which solves the problem that scientific texts based on mathematical expressions are not adapted to the traditional text retrieval system. Scharpf et al. [34] applied mathematical expressions to the document recommendation system, which annotated the variables and constants of mathematical expressions; the method disambiguates mathematical identifiers and achieves good results.

In conclusion, scientific document retrieval mainly has three methods based on text, based on mathematical expressions, and based on the fusion of mathematical expressions and text. It is difficult to describe scientific documents completely, whether it is a single mathematical expression or text, so the current scientific document retrieval mostly uses the fusion of mathematical expressions and text and uses keywords in the text, but keywords contain less information and are easy to extract inaccurately, so obtaining more text information related to mathematical expressions is also a big problem that needs to be solved. At the same time, the ontology properties of the scientific document are ignored in either way, making it difficult for the search model to meet the needs of users.

### 1.2. Contributions

**Figure 1:**
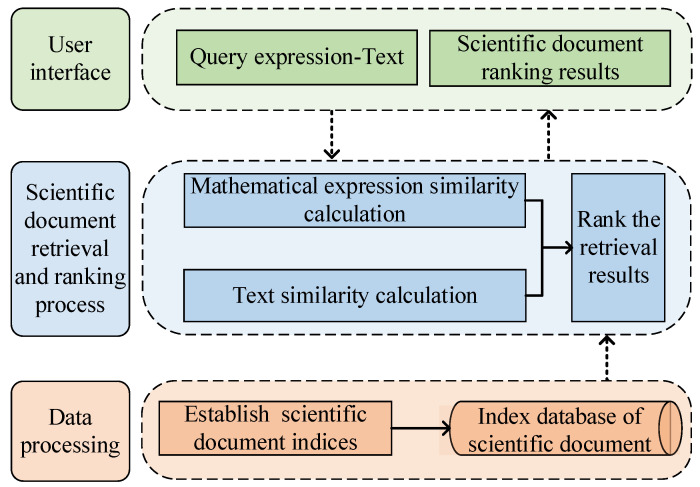
Scientific document retrieval and ranking model.

In this paper, we propose a retrieval and ranking method that integrates the content and ontology attributes of the scientific document. The ontological attributes of scientific documents are also taken into account for ranking based on mathematical expressions and text retrieval. The scientific document search model is divided into three parts, namely, the user interface, the scientific document retrieval and ranking process, and the data processing, as shown in Figure 1.

In the user interface, query expressions and text are entered and the ranked scientific documents are output. The role of data processing is to index scientific documents in a dataset and store them in a database. The scientific document retrieval and ranking process includes three parts: first, the mathematical expression similarity calculation module is used to calculate the similarity between the query expression entered by the user in LaTeX or MathML format and the candidate expression in the database; the text similarity calculation module is used to calculate the similarity between the query text and the candidate expression context, and then to synthesize the similarity of the two to obtain the scientific document retrieval results; finally, the retrieval results are ranked according to the ontology attributes of scientific documents to obtain the final ranking results.

## 2. Materials and Methods

### 2.1. Establish Scientific Document Indices

**Figure 2:**
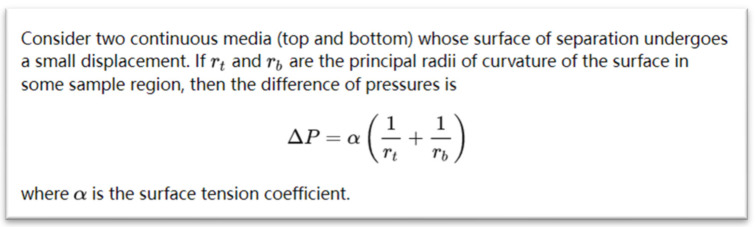
Example of a mathematical expression and its context. This document is from the open source dataset Ntcir-Mathir-Wikipedia-Corpus (http://research.nii.ac.jp/ntcir/permission/ntcir-12/perm-en-MathIR.html (accessed on 1 May 2022)).

Mathematical expressions in scientific documents have rich semantic information, and their semantics can be further interpreted by their contexts. For example, document Laplace_formula, from the Ntcir-Mathir-Wikipedia-Corpus dataset [35], its mathematical expressions and context are shown in Figure 2.

**Figure 3:**
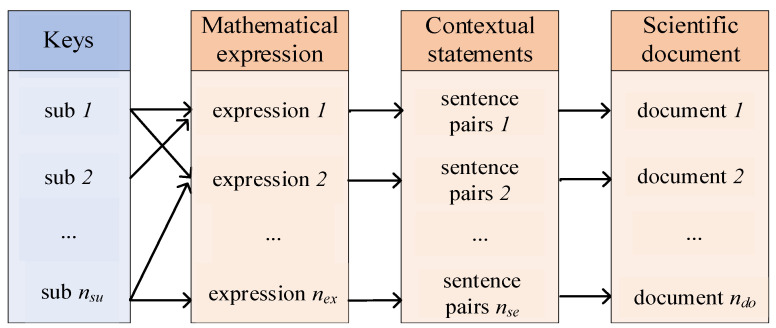
Scientific document index.

Context is closely related to mathematical expressions, so the retrieval for fused mathematical expressions and their contexts is more in line with the retrieval requirements for scientific documents. Additionally, a higher search speed is necessary for databases containing many scientific documents. The scientific document index is shown in Figure 3.

In Figure 3, the key value of the scientific document index is a sub-equation. When the user enters a query expression, the system first decomposes it into sub-equations, and by retrieving the database for sub-equations, the mathematical expression can be located directly, thus locating the expression context and the scientific document. Indexing avoids the problem of traversing the database when retrieving expressions and improves the retrieval speed of the system.

### 2.2. Mathematical Expression Similarity Calculation

In the mathematical expression similarity calculation, the user first enters a mathematical expression in LaTeX or MathML format, and then extracts the features of the mathematical expression and establishes a hesitant fuzzy set. Finally, the generalized hesitant fuzzy distance is used to calculate the similarity between the query expression and the candidate expression in the database.

#### 2.2.1. Related theories

1. Hesitant fuzzy sets

Torra [36] first proposed the concept of hesitant fuzzy sets in 2010. Hesitant fuzzy sets are extensions of fuzzy sets. The use of hesitant fuzzy sets allows experts to consider multiple evaluation attributes when making decisions, so this concept is suitable for solving the multi-attribute problem of mathematical expression matching.

Let $X=\left\{x_{1},x_{2}\cdots ,x_{n}\right\}$ be a fixed attribute set; then, $E=\left\{\langle x,h_{E}(x)\rangle |x\in X\right\}$ denotes the hesitant fuzzy set on the fixed attribute set *X*, where $h_{E}(x)$ denotes the hesitant fuzzy element (HFE). Each hesitant fuzzy element can contain one or more evaluation values, and its value range is [0, 1].

2. Hesitant fuzzy measure

If *R* and *Q* denote hesitant fuzzy sets corresponding to two samples on the same fixed attribute set $X=\left\{x_{1},x_{2},\cdots ,x_{n}\right\}$, the degree of similarity between two samples on attribute set *X* can be measured by calculating the generalized hesitant fuzzy distance between two hesitant fuzzy sets. The smaller the distance is, the greater the similarity between the two samples [37]. The generalized hesitant fuzzy distance between hesitant fuzzy sets is calculated as Equation (1):

(1) $$d_{ghn}(R,Q)={\left[\frac{1}{n}\sum_{i=1}^{n}\left(\frac{1}{l_{x_{i}}}\sum_{j=1}^{l_{x_{i}}}{\left|h_{R}^{\sigma (j)}(x_{i})-h_{Q}^{\sigma (j)}(x_{i})\right|}^{\lambda }\right)\right]}^{\frac{1}{\lambda }}$$

In Equation (1), *n* denotes the number of evaluation attributes of the hesitant fuzzy set, $l_{x_{i}}$ denotes the number of evaluation values, $h_{R}^{\sigma (j)}(x_{i})$ denotes the *j*th hesitant fuzzy element for the attributes $x_{i}$, and *λ* denotes the control parameter. When *λ* = 1, the upper distance is the hesitant standard Hamming distance; when *λ* = 2, the upper distance is the hesitant standard Euclidean distance.

#### 2.2.2. Construct Hesitant Fuzzy Sets of Mathematical Expressions

**Definition** **1.** $Q_{E}$ *denotes a query expression,* $R_{E_{K}}(k=1,2,\ldots ,nR)$*denotes the kth result of the mathematical expression retrieval, and *nR *denotes the number of result expressions.*

**Definition** **2.** *The attribute information of a mathematical expression is the four-tuple, which describes the length attribute of the original form of the mathematical expression, the length attribute of the parsing structure of the mathematical expression, the sub-attribute of the relationship between the mathematical expression and its sub, and the attribute of the number of sub-expressions; a set of hesitant fuzzy evaluation attributes of mathematical expressions are established based on the above attributes* $E_{A}=\left[A_{lenei},A_{lenec},A_{sub},A_{numsub}\right]$.

**Definition** **3.** $HFS_{Q_{E}}$*denotes the query expression hesitant fuzzy set, and*$HFS_{R_{E_{K}}}$ *denotes the result expression hesitant fuzzy set.* $h_{HFS_{Q_{}}}(E_{A_{i}})$ i∈(lenei,lenec,sub,numsub) *denotes the membership set of attributes* $A_{i}$ *for the query expression, and* $h_{HFS_{R_{K}}}^{}(E_{A_{i}})$ *denotes the membership set for the kth result expression for attribute* $A_{i}$.

**Definition** **4.** *The membership function of the mathematical expression primitive length attribute is shown in Equation (2):*

(2) $$\mu_{lenei}=\exp\left(-\frac{\left|lenei(Q_{E})-lenei(R_{E_{K}})\right|}{lenei(Q_{E})}\right)$$

*where* $lenei(Q_{E})$ *denotes the query expression primitive length and* $lenei(R_{E_{K}})$ *denotes the result expression primitive length.*

**Definition** **5.** *The skeleton extraction of mathematical expressions can eliminate the influence of variables on the mathematical expressions retrieval, so the introduction of the improved FDS* [7] *algorithm for skeleton extraction of mathematical expressions is to extract the operator information of mathematical expressions; discarding the operand information and emphasizing operator information can express mathematical expressions fully.*

The membership function of the mathematical expression parsing structure length attribute is shown in Equation (3).

(3) $$\mu_{lenec}=\exp\left(-\frac{\left|lenec(Q_{E})-lenec(R_{E_{K}})\right|}{lenec(Q_{E})}\right)$$

where $lenec(Q_{E})$ denotes the length of the query expression parsing skeleton and $lenec(R_{E_{K}})$ denotes the length of the result expression parsing skeleton.

**Definition** **6.** *When sub-expression membership is being calculated, the mathematical expression is first split into multiple sub-equations, and then the sub-expression weights are calculated according to Equation (4); the method solves the problem of retrieving not only the mathematical expression itself, but also the sub-equations.*

(4) $$\mu_{sub}=\frac{\sqrt{\frac{n_{esub}}{n_{e}}\times \frac{l_{esub}}{l_{e}}}}{\sqrt[{3}]{level}}$$

*where* $n_{e}$ *denotes the number of mathematical expression operators,* $l_{e}$ *denotes the length of the mathematical expression, and* level *denotes the lowest level of operators in the sub-equation. The mathematical expression can be expressed as* $\left\{\left(E_{sub_{1}},\mu_{sub_{1}}\right),\left(E_{sub_{2}},\mu_{sub_{2}}\right)\ldots \left(E_{sub_{n}},\mu_{sub_{n}}\right)\right\}$ *by splitting the mathematical expression and calculating the weights of the sub-equation.*

**Definition** **7.** *The membership function of the number of sub-equations attribute is shown in Equation (5):*

(5) $$\mu_{numsub}=\exp\left(-\frac{\left|numsub(Q_{E})-numsub(R_{E}{}_{{}_{K}})\right|}{numsub(Q_{E})}\right)$$

*where* $numsub(Q_{E})$ *denotes the number of sub-equations of the query expression and* $numsub(R_{E_{K}})$ *denotes the number of sub-equations of the resulting expression.*

#### 2.2.3. Mathematical Expression Matching

According to the membership calculation method of the related attribute, the hesitant fuzzy set of query expression can be expressed as $HFS_{Q_{E}}=\left[1,1,(\mu_{sub_{1}}^{Q_{E}},\mu_{sub_{2}}^{Q_{E}}\ldots \mu_{sub_{i}}^{Q_{E}}\ldots \mu_{sub_{n}}^{Q_{E}}),1\right]$. The resulting mathematical expressions hesitant fuzzy sets is shown in Table 1.

The mathematical expression matching algorithm is shown in Algorithm 1.


**Algorithm 1** Mathematical expression-matching algorithmInput: Query expression $Q_{E}$ and Result expression $R_{E_{K}}$Output: Mathematical expression similarity $sim(Q_{E},R_{E_{K}})$
1$\mu_{lenei}$ = calculatelenei($Q_{E}$, $R_{E_{K}}$);   //Evaluates the original expression length membership;2$\mu_{lenec}$ = calculatelenec($Q_{E}$, $R_{E_{K}}$);3$\mu_{numsub}$ = calculatenumsub($Q_{E}$, $R_{E_{K}}$);4for $E_{sub}^{Q_{E}}$ in $listE_{sub}^{Q_{E}}$    //Evaluates the membership of the subexpression of query expression;5$\mu_{sub_{}}^{Q_{E}}$ = calulatesub($E_{sub}^{Q_{E}}$, $Q_{E}$);6$Q_{E}$ = $\left[\left(E_{sub_{1}}^{Q_{E}},\mu_{sub_{1}}^{Q_{E}}\right),\left(E_{sub_{2}}^{Q_{E}},\mu_{sub_{2}}^{Q_{E}}\right),\ldots ,\left(E_{sub_{i}}^{Q_{E}},\mu_{sub_{i}}^{Q_{E}}\right),\ldots ,\left(E_{sub_{n}}^{Q_{E}},\mu_{sub_{n}}^{Q_{E}}\right)\right]$;7for $E_{sub}^{R_{E_{K}}}$ in $listE_{sub}^{R_{E_{K}}}$;8$\mu_{sub}^{R_{E_{K}}}$ = calculatesub($E_{sub}^{R_{E_{K}}}$, $R_{E_{K}}$);9$R_{E_{K}}$ = $\left[\left(E_{sub_{1}}^{R_{E_{K}}},\mu_{sub_{1}}^{R_{E_{K}}}\right),\left(E_{sub_{2}}^{R_{E_{K}}},\mu_{sub_{2}}^{R_{E_{K}}}\right),\ldots ,\left(E_{sub_{i}}^{R_{E_{K}}},\mu_{sub_{i}}^{R_{E_{K}}}\right),\ldots ,\left(E_{sub_{n}}^{R_{E_{K}}},\mu_{sub_{n}}^{R_{E_{K}}}\right)\right]$;10for sub in $Q_{E}$        //Resets sub-equation membership according to matching relationships;11if ($sub\in Q_{E}$ and $sub\in R_{E_{K}}$);12$\mu_{sub}^{Q_{E}}=\mu_{sub}^{Q_{E}}$; $\mu_{sub}^{R_{E_{K}}}=\mu_{sub}^{R_{E_{K}}}$;13else if ($sub\notin Q_{E}$ and $sub\in R_{E_{K}}$);14$\mu_{sub}^{Q_{E}}=0$; $\mu_{sub}^{R_{E_{K}}}=0$;15else if ($sub\in Q_{E}$ and $sub\notin R_{E_{K}}$);16$\mu_{sub}^{Q_{E}}=\mu_{sub}^{Q_{E}}$; $\mu_{sub}^{R_{E_{K}}}=0$;17else{break;};18$HFS_{Q_{E}}=\left\{1,1,(\mu_{sub_{1}}^{Q_{E}},\mu_{sub_{2}}^{Q_{E}},\ldots ,\mu_{sub_{i}}^{Q_{E}},\ldots ,\mu_{sub_{n}}^{Q_{E}}),1\right\}$;       //Build hesitant fuzzy set;19$HFS_{R_{E_{K}}}=\left\{\mu_{lenei}^{R_{E_{K}}},\mu_{lenec}^{R_{E_{K}}},(\mu_{sub_{1}}^{R_{E_{K}}},\mu_{sub_{2}}^{R_{E_{K}}},\ldots ,\mu_{sub_{i}}^{R_{E_{K}}},\ldots ,\mu_{sub_{n}}^{R_{E_{K}}}),\mu_{numsub}^{R_{E_{K}}}\right\}$;20$d(HFS_{Q_{E}},HFS_{R_{E_{K}}})=\frac{1}{4}{\sum_{i=1}^{4}\left[\frac{1}{l_{A_{i}}}\sum_{j=1}^{l_{A_{I}}}{\left|h_{HFS_{Q_{E}}}^{\sigma (j)}(Q_{E_{Ai}})-h_{HFS_{R_{E_{Ki}}}}^{\sigma (j)}(R_{E_{K_{Ai}}})\right|}^{\lambda }\right]}^{\frac{1}{\lambda }}$;21return $sim(Q_{E},R_{E_{K}})=1-d(HFS_{Q_{E}},HFS_{R_{E_{K}}})$;   //Return mathematical expression similarity.


### 2.3. Text Similarity Calculation

#### 2.3.1. Related Theories

**Figure 4:**
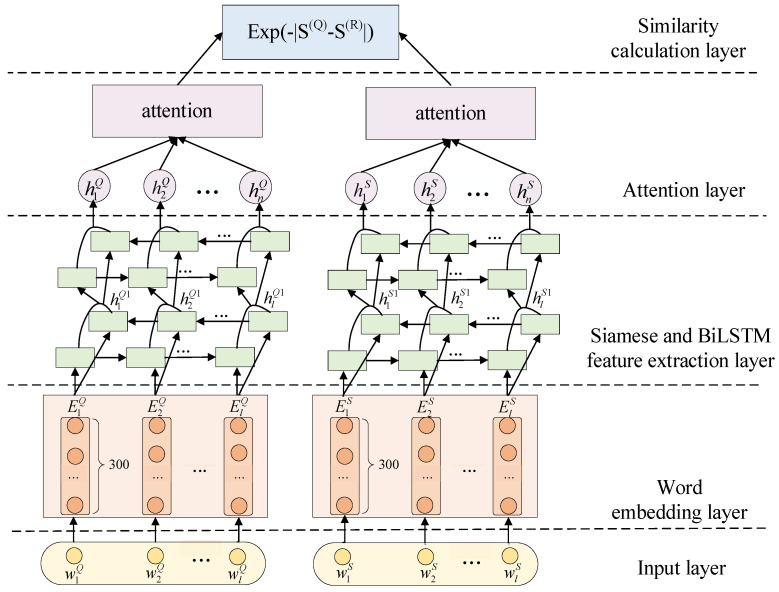
Sentence similarity calculation model.

At present, in the retrieval method based on the fusion of mathematical expressions and text, most of the keyword-based methods are used to process text, but methods based on global keyword extraction make keyword extraction inaccurate, and context-based keyword extraction is limited by fewer statements. Therefore, the use of contextual statements not only enriches the semantics of mathematical expressions and avoids the problem of unclear semantics, but also preserves the meaning of the mathematical expression context itself. In 2015, Huang et al. [38] proposed the bidirectional LSTM-CRF model to deal with sequence labeling tasks. As the bidirectional encoding feature of bidirectional LSTM is also applicable to calculating sentence similarity and solves the problem of a single LSTM encoding direction, we adopt the BiLSTM model when using sentence similarity and introduce an attention mechanism to give weight to words to distinguish between important and irrelevant parts of sentences. The sentence similarity calculation is divided into four layers, namely, the input layer, word embedding layer, Siamese and BiLSTM feature extraction layer, and attention layer and similarity calculation layer, as shown in Figure 4.

#### 2.3.2. Sentence Similarity Calculation

1. Input layer

In the input layer, the Chinese dataset first uses jieba to segment the sentence *S*, the English dataset does not need to be segmented, and then the sentence is processed, including by removing stop words and unifying the sentence length. The experiment stipulates that the maximum length of the Chinese sentence lc=20, the maximum length of the English sentence le=30, parts that exceed the length of the sentence are removed, and the short parts is completed. After the input layer, the sentence *S* can be represented as $S=[w_{1},w_{2},\ldots ,w_{i},\ldots ,w_{l}]$.

2. Word embedding layer

In the word embedding layer, the 8-million-word vector provided by Tencent AI is used by the Chinese dataset for word embedding, the 3 million common words trained based on GoogleNews’ corpus is used by the English dataset, and each word vector has 300 dimensions. After word embedding, a word vector’s sentences can be represented as a matrix of S=[l×300], where l denotes the maximum sentence length.

3. Siamese and BiLSTM feature extraction layer

In the feature extraction layer, the bi-layer stacked BiLSTM is used to extract features from the sentence to achieve bidirectional encoding of the sentence. The output of the first layer of BiLSTM acts as the input of the second layer of BiLSTM. At time *t*, the word *w* is given $\vec{h_{t}}$ by forward LSTM and $\overset{\leftarrow }{h_{t}}$ by reverse LSTM, the word vector of the word *w_t_* is expressed as $h_{t}=[\vec{h_{t}}:\overset{\leftarrow }{h_{t}}]$ by stitching the forward and backward vectors, and the sentence vector can be expressed as $S=\{[\vec{h_{1}}:\overset{\leftarrow }{h_{1}}],[\vec{h_{2}}:\overset{\leftarrow }{h_{2}}],\ldots ,[\vec{h_{n}}:\overset{\leftarrow }{h_{n}}]\}$. The word vector is spliced together to obtain the sentence vector after the BiLSTM layer feature extraction is $S=\{h_{1},h_{2},\ldots ,h_{n}\}$.

4. Attention layer

Each word contributes differently to the sentence, and the weight of the words that are important to the sentence should also be higher, so introducing attention mechanisms and assigning a higher weight to important words make the feature extraction of the sentence more effective. When the sentence is featured by BiLSTM, the word vector $h_{i}$ is obtained by performing a nonlinear transformation by using the tan*h* activation function to obtain $u_{i}$, and then the softmax function is used to obtain the weight of each word vector. The calculation formula is shown in Equation (6), and finally, the sentence vector is obtained by cumulative multiplication of Equation (7).

(6) $$\alpha_{i}=\frac{\exp(u_{i})}{\sum_{j=1}^{n}\exp(u_{j})}$$

(7) $$S=\sum_{i=1}^{n}\alpha_{i}\cdot h_{i}$$

5. Similarity calculation layer

In the experiment, the Manhattan distance is chosen as the calculation of sentence vector similarity with the value of [0, 1], which is calculated as shown in Equation (8).

(8) $$sim(S^{\left(Q\right)},S^{\left(R\right)})=\exp(-\left|S^{\left(Q\right)}-S^{\left(R\right)}\right|)$$

### 2.4. Rank the Retrieval Results

In the traditional scientific document retrieval mainly based on mathematical expressions, usually only the internal information of documents is considered, and ontological information is often missing. However, the user’s demand for scientific document retrieval not only remains on the content, such as expressions and text, but also pays attention to the category of scientific documents, and other document ontology information, based on the user’s ranking needs for scientific documents, rank the retrieval results on the basis of expression and text retrieval so that the recalled scientific documents can better meet user needs.

**Definition** **8.** *Scientific document matching results*$list_{MD}=\{doc_{1}^{1},\ldots ,doc_{i}^{j},\ldots ,doc_{nd}^{c}\}$*, where* $doc_{i}^{j}$ *denotes the ith scientific document, j denotes the document’s category number, and nd denotes the number of matching results. c denotes the number of categories,*$list_{CD}$ *denotes the list of scientific documents classified according to the category,* $list_{PD}$ *denotes the list of scientific documents ranked according to the popularity of the category, and* $list_{SD}$ *denotes the final ranking list of the scientific documents.*

First, the scientific document matching results $list_{MD}$ are divided by categories, and the list of scientific documents can be expressed as $list_{CD}=\{U_{D}^{1},\ldots ,U_{D}^{j},\ldots ,U_{D}^{c}\}$, where $U_{D}^{j}$ denotes the collection of scientific documents with category number *j*.

For users, the purpose of the document is likely to belong to the same category; for example, after the user enters the relevant physical formulas and statements, the purpose of the document most likely belongs to the physical class, rather than other categories, so the popularity of the category as the ranking basis of scientific documents is very reasonable. The proportion of scientific documents in each category is calculated; the larger the proportion of documents in a category is, the more popular the category is in the scientific document matching results and the greater the probability that the users demand for scientific documents belongs to this category. The popularity $P_{cate}^{j}$ of category *j* is calculated as shown in Equation (9).

(9) $$P_{cate}^{j}=\frac{count(U_{D}^{j})}{\sum_{i=1}^{c}count(U_{D}^{i})}$$

where $count(U_{D}^{j})$ is the number of scientific documents in category *j*. The scientific document is ranked in descending order by category popularity, and the results can be expressed as $list_{PD}=\{U_{D}^{j},\ldots ,U_{D}^{c},\ldots ,U_{D}^{1}\}$. $U_{D}^{j}$ is the most popular and $U_{D}^{1}$ is the least popular.

Additionally, users always want to recall the latest documents for more cutting-edge content, so it is also important to introduce the year of publication into the ranking of documents. The scientific documents in each category in $list_{PD}$ are ranked by the year of publication, and the final ranking result of the scientific documents is $list_{SD}=\{doc_{i}^{j},doc_{k}^{j},\ldots ,doc_{i}^{c},\ldots ,doc_{nd}^{c},\ldots ,doc_{2}^{1},doc_{1}^{1}\}$, where $doc_{i}^{j}$ is the most popular scientific document with the most recent publication year.

The ranking algorithm is shown in Algorithm 2.
**Algorithm 2** Scientific document ranking algorithmInput: Scientific document retrieval results $list_{MD}$Output: Scientific document ranking results $list_{SD}$1//Define a category dictionary (the key is a category, and the content is a scientific document)2Dictionary < string, List < string >> $Dic_{doc}$ = new Dictionary < string, List < string >> ();3for MD in $list_{MD}$        //Traverse the retrieval results of scientific document4{5if (!$Dic_{docs}$.ContainsKey(MD.category))//Add it if the category does not exist in the dictionary6$Dic_{docs}$. Add(MD.category, MD);7else        //Otherwise, add the scientific document to an existing category8{9List < string > Tvalue = $Dic_{docs}$ [category];  //Stores dictionary values temporarily10Tvalue. Add(doc);11$Dic_{docs}$ [category] = Tvalue;12}13}14//Rank scientific documents in descending order by category popularity15$Dic_{docs}$ = $Dic_{docs}$ Sorting by $Dic_{docs}$.Values. Count DESE;16//Sort the scientific documents in each category in descending order and return the results17for (var $Dic_{doc}$ in $Dic_{docs}$)18{19return $list_{SD}$ Sorting by doc.postdate DESE;20}

## 3. Results and Discussion

Ntcir-Mathir-Wikipedia-Corpus is considered for this research work, which contains 31,740 documents and 529,621 expressions; this dataset includes only English documents. Thus, we introduce 10,371 Chinese documents [28], including 139,586 mathematical expressions, to expand the dataset.

### 3.1. Mathematical Expression Matching Results

The experiment selects 10 mathematical expressions and their related statements, as shown in Table 2, as queries. These 10 expressions and their query statements contain common operation symbols and meanings when using mathematical expressions.

MAP_*k* (mean average precision_*k*) is the average of AP_*k*, which is calculated as shown in Equation (10).

(10) $$\mathrm{MAP}\_k=\frac{1}{Q}\sum_{q=1}^{Q}\mathrm{AP}\_k(q)$$

where *Q* denotes the number of queries and AP_*k*(*q*) denotes the AP_*k* value of the *q*th query, which is calculated as shown in Equation (11).

(11) $$\mathrm{AP}\_k=\frac{1}{K}\sum_{k=1}^{K}\frac{k}{\mathrm{position}(k)}$$

The MAP_*k* values of mathematical expressions in the English dataset and the Chinese dataset are shown in Table 3. As the data in the table show, the MAP_5 of mathematical expressions is close to MAP_10, because the mathematical expression retrieval method used in this paper focuses on the sub-equation and the operator so that the recalled expression distribution is more uniform. The MAP_15 value is lower because many expressions are the same but the representation differs, so the similarity differs, and the low number of similar expressions contained in the database is one of the reasons for the low MAP_15 value.

### 3.2. Ranking Results of Scientific Documents

The NDCG (normalized discounted cumulative gain) is a measure and evaluation of search results; the NDCG is calculated as shown in Equation (12).

(12) $$\mathrm{NDCG}@k=\frac{\mathrm{DCG}@k}{\mathrm{IDCG}@k}$$

where the IDCG (ideal discounted cumulative gain) is the DCG (discounted cumulative gain) value in the ideal situation, *k* is the first *k* term of the query results, and the DCG and IDCG calculation formulas are shown in Equations (13) and (14), respectively.

(13) $$\mathrm{DCG}@k=rel_{i}+\sum_{i=2}^{k}\frac{rel_{i}}{\log_{2}(i+1)}$$

(14) $$\mathrm{IDCG}@k=\sum_{i=1}^{\left|REL\right|}\frac{rel_{i}}{\log_{2}(i+1)}$$

where $rel_{i}$ denotes the relevance score. According to the expert score, the retrieval results are divided into three cases (namely, similar, partially similar, and not similar) and given a score of 3, 2, and 1, respectively, as the relevance score of the search results; $\log_{2}(i+1)$ is a discount factor.

**Figure 5:**
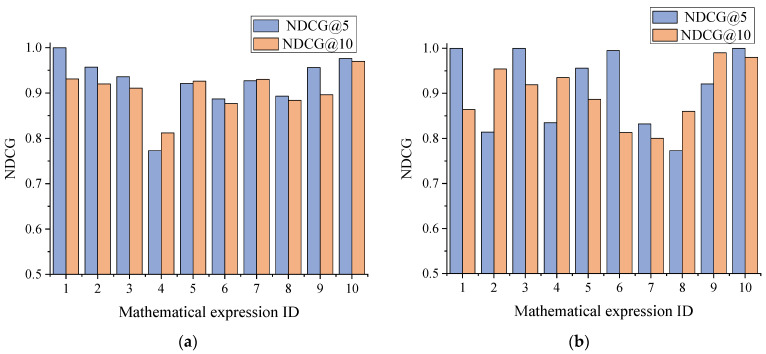
NDCG@5 and NDCG@10 values. (**a**) Chinese dataset. (**b**) English dataset.

Figure 5 lists the NDCG@5 and NDCG@10 values of the ranking of scientific documents for the Chinese dataset and the English dataset, respectively. As the figures show, the NDCG@5 values of the Chinese and English scientific documents are higher than the NDCG@10 values, and the NDCG values of the scientific documents are almost above 0.8, thus proving that the scientific document retrieval method adopted in this paper is more reasonable.

Table 4 shows the NDCG@*k* and MAP_*k* values of the scientific document retrieval and ranking results of the Chinese and English datasets in different methods, where NDCG@5, NDCG@10, and MAP_5, MAP_10 are taken as the average of the 10 query results in Table 2. It can be found that the retrieval of scientific documents can be achieved when using mathematical expressions or text alone, and the retrieval effect based on mathematical expressions is better because the retrieval of mathematical expressions is more regular and accurate compared with the text. It can be found that the retrieval effect is further improved after fusing expressions with text for scientific document content retrieval, and the retrieval results can further satisfy users after introducing the ontology attributes of scientific documents; therefore, the results are optimal.

**Figure 6:**
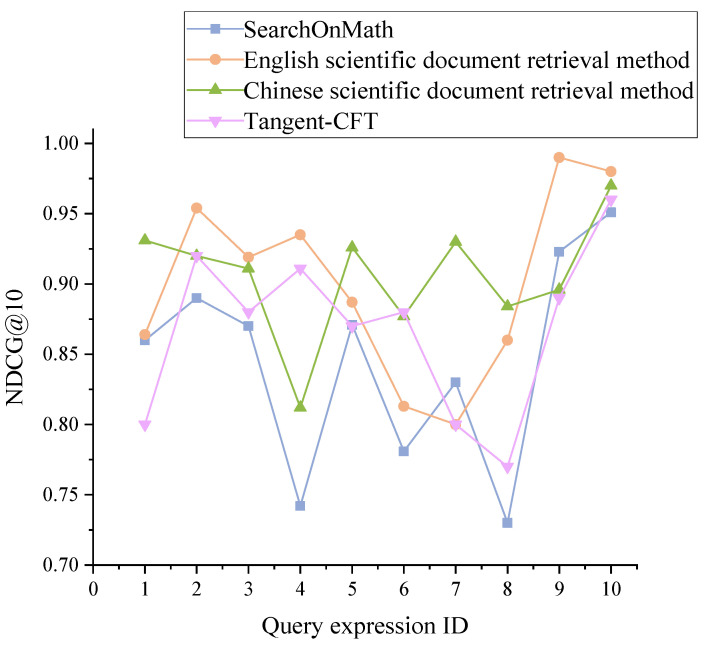
Comparison of NDCG@10 values of other methods with those of our method.

SearchOnMath [39] is a system proposed by Oliveira et al. for retrieving scientific documents and Wikipedia English content by using mathematical expressions or keywords, including 1905358 mathematical expressions, and users can retrieve relevant content by entering mathematical expressions or keywords. Tangent-CFT [10] is an open-source mathematical expression embedding model proposed by Mansouri et al.; this model integrates OPTs and SLTs to capture expression content and expression structure, respectively, and finally uses fastText to generate formula embedding. The mathematical expressions and statements in Table 2 are entered into our system, SearchOnMath and Tangent-CFT, and Figure 6 compares the NDCG@10 values of the SearchOnMath method, the Chinese scientific document retrieval method, the English scientific document retrieval method, and the Tangent-CFT method.

As Figure 6 shows, the NDCG@10 values of the Chinese and English scientific document methods are basically higher than those of the comparison method because the method proposed in this paper introduces the ontological attributes of scientific documents while considering the content of scientific documents, and then making the ranking results better meet the needs of users. In contrast to the SearchOnMath method, this method effectively avoids unreasonable sorting problems caused by using mathematical expressions or text alone. In terms of text processing, introducing contextual sentences avoids inaccurate retrieval results caused by inaccurate keyword extraction. Additionally, the Tangent-CFT method starts only from the mathematical expression itself and does not pay attention to the global information of scientific documents, so the NDCG@10 value of this method is lower than that of our system.

## 4. Conclusions

Based on the scientific document retrieval model incorporating mathematical expressions with related texts, a retrieval and ranking model combining scientific document content and ontology attributes is proposed; the model first decomposes the mathematical expression into sub-equations; then, the hesitant fuzzy set is built according to the mathematical expression with sub-equation membership, and finally calculates the generalized hesitant fuzzy distance to obtain the similarity of mathematical expressions. In terms of text matching, the mathematical expression context statement is extracted, and then the sentence similarity is calculated by combining BiLSTM with the attention mechanism. Finally, the mathematical expression similarity and sentence similarity are synthesized to obtain the retrieval results of the scientific document. This method solves the problem of single retrieval modes relying only on mathematical expression or text, and the use of sentences can better retain the original information of the context and avoid inaccurate keyword extraction. Additionally, document categories are extracted from scientific document ontology features and sorted according to their popularity, and then the documents in the category are ranked by year of publication to obtain the final ranking results. The experimental results show that the scientific document retrieval and ranking method combining content and ontology features better meets user needs.

Future work:

- While searching scientific documents by using mathematical expressions, we will continue to explore the method of extracting related text information to improve the connection between expressions and related texts.
- We will consider the ontological characteristics of scientific documents from multiple angles and extract more ontological information from documents to make the ranking of scientific documents more reasonable.

## Figures and Tables

**Table 1:** Result expressions hesitant fuzzy sets.

	Membership	Original Expression Length	Parsing Expression Length	Sub-Expression	The Number of Sub-Expressions
ID					
1		$\mu_{lenei}^{R_{E_{1}}}$	$\mu_{lenec}^{R_{E_{1}}}$	$\mu_{sub_{1}}^{R_{E_{1}}},\mu_{sub_{2}}^{R_{E_{1}}}\ldots \mu_{sub_{i}}^{R_{E_{1}}}\ldots \mu_{sub_{n}}^{R_{E_{1}}}$	$\mu_{numsub}^{R_{E_{1}}}$
2		$\mu_{lenei}^{R_{E_{2}}}$	$\mu_{lenec}^{R_{E_{2}}}$	$\mu_{sub_{1}}^{R_{E_{2}}},\mu_{sub_{2}}^{R_{E_{2}}}\ldots \mu_{sub_{i}}^{R_{E_{2}}}\ldots \mu_{sub_{n}}^{R_{E_{2}}}$	$\mu_{numsub}^{R_{E_{2}}}$
…		…	…	…	…
k		$\mu_{lenei}^{R_{E_{K}}}$	$\mu_{lenec}^{R_{E_{K}}}$	$\mu_{sub_{1}}^{R_{E_{K}}},\mu_{sub_{2}}^{R_{E_{K}}}\ldots \mu_{sub_{i}}^{R_{E_{K}}}\ldots \mu_{sub_{n}}^{R_{E_{K}}}$	$\mu_{numsub}^{R_{E_{K}}}$
…		…	…	…	…
nR		$\mu_{lenei}^{R_{E_{nR}}}$	$\mu_{lenec}^{R_{E_{nR}}}$	$\mu_{sub_{1}}^{R_{E_{nR}}},\mu_{sub_{2}}^{R_{E_{nR}}}\ldots \mu_{sub_{i}}^{R_{E_{nR}}}\ldots \mu_{sub_{n}}^{R_{E_{nR}}}$	$\mu_{numsub}^{R_{E_{nR}}}$

**Table 2:** Query expressions and related statements.

ID	Query Expressions	Query Statements
1	$\bar{x}=\frac{1}{n}\sum_{i=1}^{n}x_{i}$	Given *n* samples data, the sample mean is
2	$x=\frac{-b\pm \sqrt{b^{2}-4ac}}{2a}$	Two solutions of any quadratic polynomial can be expressed as follows
3	$\sin^{2}\theta +\cos^{2}\theta =1$	The basic relationship between sines and cosines is called the Pythagorean theorem
4	$\underset{x\to a}{\lim}f(x)=L$	means that *ƒ*(*x*) can be made as close as desired to *L* by making *x* close enough but not equal to *a*
5	$\int_{a}^{b}f(x)dx$	The definite integral *ƒ*(*x*) over an interval [a, b] can be written as
6	$f(x)=ax^{2}+bx+c$	The quadratic polynomial of *ƒ*(*x*) can be expressed as follows
7	$E=mc^{2}$	The relationship between mass and energy in special relativity is as follows
8	O(*n*^2^)	The time complexity of the algorithm is
9	$r_{s}=\frac{2GM}{c^{2}}$	*G* denotes Newton’s gravitational constant, *m* denotes the mass of the electron, and *c* denotes the speed of light
10	$E=\frac{1}{2}mv^{2}$	The kinetic energy formula is expressed as follows

**Table 3:** MAP_*k* values of mathematical expressions.

Dataset Name	MAP_5	MAP_10	MAP_15
English dataset	0.831	0.815	0.765
Chinese dataset	0.823	0.802	0.712

**Table 4:** MAP_*k* and NDCG@*k* at Top-*k* Results.

Method	NDCG@5		NDCG@10		MAP_5		MAP_10	
	English	Chinese	English	Chinese	English	Chinese	English	Chinese
Math expressions	0.830	0.851	0.707	0.754	0.823	0.796	0.703	0.685
Text	0.750	0.799	0.685	0.727	0.803	0.763	0.690	0.703
Content	0.892	0.887	0.816	0.799	0.850	0.830	0.800	0.754
Content and ontology	0.913	0.923	0.900	0.906	0.892	0.874	0.875	0.854

## Data Availability

The data that support the findings of this study are available upon request.
